# The role of teichoic acids of bifidobacteria in driving the interaction with the human host

**DOI:** 10.3389/fmicb.2025.1616397

**Published:** 2025-07-10

**Authors:** Giulia Longhi, Laura Maria Vergna, Gabriele Andrea Lugli, Massimiliano Giovanni Bianchi, Chiara Tarracchini, Christian Milani, Francesca Turroni, Ovidio Bussolati, Douwe van Sinderen, Marco Ventura

**Affiliations:** ^1^Laboratory of Probiogenomics, Department of Chemistry, Life Sciences, and Environmental Sustainability, University of Parma, Parma, Italy; ^2^Microbiome Research Hub, University of Parma, Parma, Italy; ^3^Laboratory of General Pathology, Department of Medicine and Surgery, University of Parma, Parma, Italy; ^4^APC Microbiome Institute and School of Microbiology, Bioscience Institute, National University of Ireland, Cork, Ireland

**Keywords:** *B. bifidum* PRL2010, bifidobacteria-human cell line interaction, transcriptomics, probiotics, extracellular structures

## Abstract

An *in silico* survey carried out in this study, performed on more than 3,000 publicly available bifidobacterial genome sequences, revealed clear differences in genes predicted to be involved in teichoic acid (TA) biosynthesis at inter-species level, indicating that only a small number of species possess a specific cluster for the production and assembly of these cell envelope-associated structures. Among these, *Bifidobacterium bifidum* represents a key member of the human gut microbiota at the early stages of life. Here, we show differences in the transcriptomic profiles of genes predicted to be involved in TA biosynthesis of three different strains of *B. bifidum* PRL2010 following exposure to human intestinal epithelial cells, suggesting a strain-specific and host-mediated molecular response. Notably, such data were further corroborated by *in vitro* experiments with isogenic derivatives of *B. bifidum* PRL2010 carrying mutations located in one of the genetic loci putatively responsible for TA synthesis, which suggests that TAs of PRL2010 are involved in establishing microbe-host cross talk by modulating adhesion to epithelial intestinal cells, as well as by affecting the interaction with human macrophages. We furthermore revealed that some of the key genes implicated in TA synthesis by PRL2010 are overexpressed under *in vivo* conditions when the microorganism is colonizing the murine gut, which further confirms a host modulatory effect on the production of these extracellular structures in bifidobacteria.

## Introduction

The human gut microbiota represents one of the most complex and dense bacterial populations in the biosphere and has attracted substantial scientific interest in recent decades, mainly due to the widely recognized impact that this microbial community exerts on human health (Del Chierico et al., 2022; Fan and Pedersen, 2021; Ruiz-Saavedra et al., 2024; Tremaroli and Backhed, 2012). Amongst the multitude of microorganisms populating the human gut, members of the genus *Bifidobacterium* represent one of the most studied as they are considered to be among the first colonizers of this ecological niche (Martin et al., 2023; Turroni et al., 2009). Indeed, bifidobacteria are believed to exhibit their health-promoting effects from the early stages of host life, supporting host immune system development, promoting pathogen exclusion and limiting their proliferation, preventing or ameliorating gastrointestinal diseases such as inflammatory bowel disease and irritable bowel syndrome (Duranti et al., 2016; Henrick et al., 2021; Muñoz et al., 2011; Rutsch et al., 2020; Spor et al., 2011). These beneficial activities are believed to be mediated by various bioactive microbial metabolites, including conjugated linoleic acid, indole lactic acid, short chain fatty acids and vitamins, which can be favorably exploited by the host as immune signals and/or (micro)nutrients (Alessandri et al., 2023; Bottacini et al., 2014; D’Aimmo et al., 2024; Khromova et al., 2022; LeBlanc et al., 2013; Sakurai et al., 2019). However, although it has been claimed that the relative abundance of bifidobacteria in the human gut decreases with host aging, when the gut ecosystem evolves from an “infant-” to an “adult-like” gut microbiota, they remain relatively stable and persist during the entire life span of the host, decreasing only at old age (Arboleya et al., 2016; Milani et al., 2017a; Odamaki et al., 2016; Reuter, 2001; Rizzo et al., 2024). The ability of bifidobacteria to colonize and persist in the intestinal environment has been attributed to their genetic arsenal, including a wide variety of genes encoding enzymes involved in carbohydrate metabolism, as well as genetic sequences predicted to encode the biosynthetic machinery for extracellular structures, including exopolysaccharides and pili, which promote bifidobacterial attachment to the intestinal epithelial cells, and interaction with the host and other commensal microorganisms (Alessandri et al., 2021; Fanning et al., 2012; Gutierrez et al., 2023; Hidalgo-Cantabrana et al., 2014a; Kelly et al., 2021; Kline et al., 2009; Lebeer et al., 2010; Milani et al., 2017c). However, while pili and exopolysaccharides have been extensively studied and molecularly characterized in various bacterial species residing in the human intestine (Jurášková et al., 2022; Ligthart et al., 2020; Millen et al., 2023), very little information is currently available concerning teichoic acids (TAs) produced by bifidobacteria. TAs are phosphate-rich glycopolymers typically found in the cell wall of Gram-positive bacteria, where they contribute to cell envelope architecture and are involved in key processes such as adhesion, immune modulation, and bacterial physiology. Despite being extensively studied in model organisms such as *Staphylococcus aureus* and *Bacillus subtilis*, the presence and functional relevance of TAs in the genus *Bifidobacterium* has remained largely unexplored (Rao et al., 2007; Smith et al., 2011). Only a small number of publications have described bifidobacterial TAs and their potential role in mediating interactions with a host and the surrounding environment. For instance, TAs from bifidobacteria have been shown to alleviate the chemotherapeutic side effects of 5-fluorouracil via immunomodulatory mechanisms (Guo et al., 2015) and to act as a lipid modulator with fat-reducing properties (Balaguer et al., 2022). However, while it has been reported that bifidobacteria produce atypical teichoic acids, constituted by lipoglycans (Balaguer et al., 2022; Fischer et al., 1987), very little is known about the overall genetic diversity underlying TA biosynthesis in the genus *Bifidobacterium* and the biological role/s of TAs in terms of establishing a molecular dialogue with the host. Given the emerging role of TAs as key mediators in the molecular dialogue between microbes and the host, elucidating their function in bifidobacteria could yield important insights into mechanisms supporting immune and intestinal homeostasis. From a biotechnological standpoint, understanding TA biosynthesis and function may also help identify microbial traits linked to host adaptation and resilience, advancing our knowledge of how beneficial bacteria influence gut health. In the current study, we suggest that TA biosynthesis is strain-specific and responsive to host-mediated stimuli, and that TAs contribute to cross-talk between bifidobacteria and their host. The distribution of TA-associated genes across the *Bifidobacterium* genus was assessed by establishing a comprehensive database of bifidobacterial genes implicated in TA biosynthesis. We then assessed the transcriptional response of selected *B. bifidum* strains following exposure to human epithelial cells, identifying *B. bifidum* PRL2010 as a representative strain for further investigation. Subsequent *in vitro* studies were performed involving three isogenic PRL2010 mutants, each harboring a mutation in a specific TA-associated gene, to assess the role of TAs in mediating interactions with intestinal epithelial and immune cells. These findings were further supported by data from an *in vivo* trial in which PRL2010 was administered to a rodent model, revealing enhanced expression of selected TA-biosynthesis genes during gut colonization, suggesting host-mediated regulation of these cell surface structures.

## Materials and methods

### Bifidobacterial genome selection and prediction of teichoic acid homologs

**FIGURE 1:**
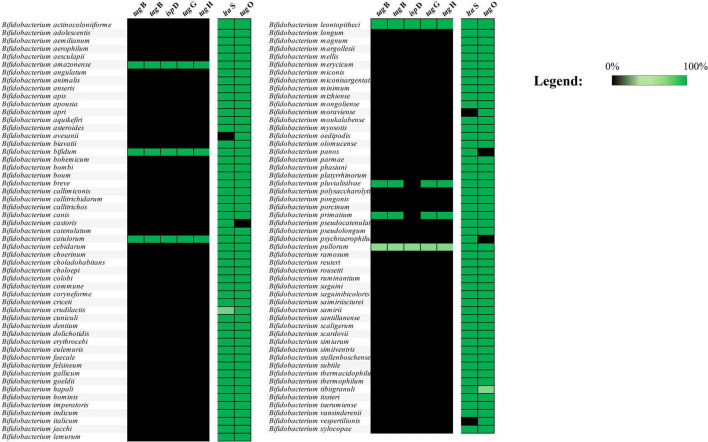
Distribution of TA-associated genes among bifidobacterial species. The image displays the distribution of TA-associated genes present in the 3,040 genomes considered in the analysis. Genes belonging to the *tag* cluster are found only in *B. amazonense, B. bifidum, B. catulorum, B. leontopitheci, B. pluvialisilvae*, *B. primatium*, and *B. pullorum* species.

Publicly available genomes (complete and draft genome sequences), encompassing all currently characterized bifidobacterial species, were retrieved in July 2024 from the NCBI public database, resulting in a dataset consisting of 3,040 bifidobacterial RefSeq genome sequences. The PGAP software of NCBI was used to predict and define genes as well as to functionally annotate the corresponding protein coding sequences. The prediction of homologs for genes associated with teichoic acid biosynthesis was first performed on the deduced proteome of the 107 Type Strains of the genus *Bifidobacterium* to assess their sequence variability. The putative TA biosynthetic gene identification was performed using sequences of genes with a deduced or proven function in TA biosynthesis in *B. bifidum* PRL2010 as well as in other actinobacteria, i.e., belonging to the *Streptomyces, Corynebacterium*, and *Mycobacterium* genera (Colagiorgi et al., 2015), employing a BLASTp analysis with an e-value of 1 × 10^–20^ (Supplementary Table S1). Each identified homolog was manually curated to eliminate false positives, which were subsequently discarded. This manual editing focused on identifying and selecting gene clusters associated with conserved protein domain families involved in TA biosynthesis. For this purpose, a hmmscan analysis was conducted using a stringent e-value threshold of 1 × 10^–50^. Genes within the same cluster were selected if they were separated by no more than three genes, with the exception of *lta*S and *tag*O. Following this, the deduced proteomes of 3,040 RefSeq bifidobacterial genomes were screened for protein homologs related to TA biosynthesis functions. The distribution of these homologs across species was analyzed using the same approach, applying a sequence identity cutoff of 50%. Then, the deduced proteome of each RefSeq bifidobacterial genome was screened for the presence of protein homologs of TA biosynthesis functions to explore their distribution among each species. A prevalence heatmap was generated using the information obtained by a BLASTp analysis with an e-value of 1 × 10^–50^ (Figure 1).

### Bifidobacterial growth conditions.

Bifidobacterial strains used (Table 1) were routinely grown at 37°C under anaerobic conditions (2.99% H_2_, 17.01% CO_2_, and 80% N_2_) (Concept 400, Ruskinn) in the de Man-Rogosa-Sharpe (MRS) broth (Sharlau Chemie, Barcelona, Spain) supplemented with 0.05% (wt/vol) L-cysteine HCl (Merk, Germany).

**TABLE 1:** Bifidobacterial strains exposed to Caco-2/HT29-MTX human cell monolayer.

Bifidobacterial species	Strain
*B. bifidum*	PRL2010
	LMG 11041
	324B

### Human cell line culture

Human colorectal adenocarcinoma-derived Caco-2 cells (purchased from ATCC) and human colon carcinoma-derived mucin-secreting goblet HT29-MTX cells (kindly provided by Prof. Antonietta Baldi, University of Milan) were cultured in Minimum Essential medium (MEM) and Dulbecco’s Modified Eagle medium (DMEM) with high glucose (4.5 g/L) and 10 mM sodium pyruvate, respectively, as previously described (Alessandri et al., 2023; Bianchi et al., 2019; Fontana et al., 2022; Tarracchini et al., 2023). Both media were supplemented with 10% Fetal Bovine Serum (FBS), 2 mM glutamine, 100 μg/mL streptomycin, and 100 U/mL penicillin. Cultures were maintained at 5% CO_2_ at 37°C and passaged three times a week. Subsequently, a mixed suspension of Caco-2 and HT29-MTX cells (7:3) was seeded in DMEM supplemented with 10% FBS at a density of ≈10^5^ cells/cm^2^ into cell culture inserts with membrane filters (pore size 0.4 μm) for Falcon 24-well-multitrays (Becton, Dickinson & Company, Franklin Lakes, NJ, United States), and cultured for 21 days with a medium replacement every 3 days until a tight monolayer was formed (TEER > 600 Ω⋅cm^2^).

### Bifidobacterial co-culture with human cell lines

Twenty-one days following the seeding event of the Caco2/HT29-MTX human cell line, the cultivation medium of the 24-well plates was replaced with fresh antibiotic-free DMEM. Subsequently, each bifidobacterial strain used in this study (Table 1) was individually added to a human cell line monolayer-containing well at a final concentration of 10^8^ cells/mL, as previously described (Fontana et al., 2022; Serafini et al., 2013). The 24-well plates were subsequently incubated at 5% CO_2_ at 37°C. After 4 h of incubation, the supernatant containing the bacterial cells was carefully aspirated, centrifuged to collect the bacterial pellet and conserved in RNA later. The adherent human cells remaining on the plate were collected by scraping the well surface directly in RNA later and similarly preserved at −80°C until further processing.

For this human cell line experiment, all bifidobacterial strains were grown in MRS broth under anaerobic conditions at 37°C. Once the exponential growth phase (0.6 < OD600 nm < 0.8) had been reached, bifidobacterial cells were enumerated using the Thoma cell counting chamber (Herka), diluted to reach a final concentration of 1 × 10^8^ cells/mL, washed in Phosphate-Buffered Saline (PBS), re-suspended in 400 μL of antibiotic-free DMEM, and then added to the Caco-2/HT29-MTX cell line monolayer. Bifidobacterial strains, re-suspended in DMEM and maintained under the same incubation conditions in 24-well plates without any contact with human cell lines, were used as bacterial control sample.

### RNA extraction

Total RNA from each bifidobacterial strain exposed to the Caco-2/HT29-MTX cell monolayer was isolated as previously described (Milani et al., 2020; Turroni et al., 2016). Briefly, bifidobacterial cell pellets were resuspended in 1 mL of QIAZOL (Qiagen, United Kingdom) and placed in a tube containing 0.8 g of glass beads (diameter of 106 μm; Merk, Germany). Cells were lysed by alternating 2 min of stirring the mix on a bead beater with 2 min of static cooling on ice for three times. Lysed cells were centrifuged at 12,000 rpm for 15 min, and the upper phase was recovered. Bacterial RNA was subsequently purified using the RNeasy Mini Kit (Qiagen, Germany) following the manufacturer’s instruction.

### Bacterial RNA sequencing analysis

The quality of the RNA was verified by employing a Tape station 2200 (Agilent Technologies, United States). RNA concentration and purity were evaluated using a spectrophotometer (Eppendorf, Germany). For RNA sequencing, an aliquot from 100 ng to 1 μg of extracted RNA was treated to remove rRNA using the QIAseq FastSelect—5 S/16 S/23 S following the manufacturer’s instructions (Qiagen, Germany). The level of rRNA depletion was checked using a Tape station 2200 (Agilent Technologies, United States). Subsequently, a transcriptome library was constructed using the TruSeq Standard mRNA Sample preparation kit (Illumina, San Diego, United States). Samples were loaded into a NextSeq high output v2.5 kit (150 cycles, single end) (Illumina) following the technical support guide. The obtained reads were filtered to remove low-quality reads (minimum mean quality of 20 and minimum length of 150 bp), as well as any remaining ribosomal locus-encompassing reads using the METAnnotatorX2 pipeline (Milani et al., 2021). Subsequently, the retained reads were aligned to the specific reference genome sequence through Bowtie2 software (Langdon, 2015), while quality of the alignments was assessed through Picard software toll (version 2.26.11)^1^. Furthermore, quantification of reads mapped to individual transcripts was achieved through htseq-counts script of HTSeq software in “union” mode (Anders et al., 2015). Raw counts were subsequently normalized using CPM (Counts per million mapped reads) for filtering genes with low counts (CPM < 1) and TMM (Trimmed Mean of *M*-values) for statistically robust differential gene expression analysis through the EdgeR package (Robinson et al., 2010). Expression differences for each gene were calculated as log_2_ fold change (logFC) of average expression between the control (no contact between human cell lines and bacterial strains) and treated samples (contact between human cell lines and bifidobacterial strains). For each comparison, a statistical significance (*p*-value) was calculated. Each experiment was conducted in triplicate.

### Plasmid manipulation

Plasmid pFREM30 (Rizzo et al., 2024) was used as a suicide vector for mutagenesis of genes encoding proteins putatively involved in TA biosynthesis, i.e., BBPR_1314, BBPR_0702, BBPR_0699. *Escherichia coli* EC101 (Law et al., 1995), used as a host strain for the propagation of the pFREM30, was cultivated at 37°C in LB medium (Luria Bertani, Scharlab, Spain) supplemented with chloramphenicol at a final concentration of 25 μg/mL To obtain the plasmids for mutagenesis, i.e., pFREM30-0699, pFREM30-0702, pFREM30- 1314, internal regions of the to-targeted genes were amplified by PCR from chromosomal *B. bifidum* PRL2010 DNA (GenElute Bacterial Genomic DNA kit, Sigma, Germany) using Taq polymerase, and the primers listed below. The sections of the genes considered represent bases 766 until 1,064 of BBPR_0699 (encompassing 1,887 bases), bases 336 until 620 of BBPR_0702 (encompassing 717 bases) and bases 314 until 699 of BBPR_1314 (encompassing 2,214 bases). Plasmid DNA was isolated from *E. coli* using the GeneJET Plasmid Maxiprep Kit (Thermo Fisher Scientific, United States). The amplicons and plasmids were digested with *Apa*LI and *Xho*I, ligated, and introduced into *E. coli* EC101 as previously reported (Hanahan et al., 1991). To select for transformants, the manipulated cells were plated on LB supplemented with 25 μg/mL chloramphenicol, and the colonies were screened for the presence of the expected plasmid construct by colony PCR, while the genetic integrity was then validated by sequencing (Eurofins, Ebersberg, Germany). The primers used are listed in Table 2.

**TABLE 2:** Primers for cloning of TA-associated genes in pFREM30 and for PRL2010 mutant screening.

Target	Primer name	Sequence 5′–3′
Cloning TA genes	1314_Fw	AATAATGTGCACGCGTCAGCCAGTCCGG
	1314_Rv	AATAATCTCGAGGACATGGTTGAGCACCAC
	699_Fw	AATAATGTGCACCGCTTCGACATTCGTGC
	699_Rv	AATAATCTCGAGCGACGCAGGTGCTCG
	702_Fw	AATAATGTGCACGCTGATCGACCAGTGGAC
	702_Rv	AATAATCTCGAGCCTTCCACCTCGTACAGT
Colony PCR screening	New_MCS1	CGAATCGCCAACGTTTTC
	LMV_702_Fw	CCGCGGATTCGGTCGTC
	LMV_702_Rv	CGATGGCGTGGTGATCTTG
	LMV_1314_Fw	GTTCGACATGGCGGCG
	LMV_1314_Rv	GGATCATGCGACCATGC
	LMV_0699_Fw	GGAGGCGTTGCAAAGCC
	LMV_0699_Rv	CCTGGGCATGCCTGTGG

### Mutants of *B. bifidum* PRL2010

*B. bifidum* PRL2010 mutants were obtained and cultivated as previously described (Rizzo et al., 2024). Potential mutants were screened by colony PCR using the forward primers annealing to the chromosomal gene outside of the gene target region (LMV_702_Fw, LMV_1314_Fw, LMV_0699_Fw), and new_MCS1, which annealed to the (integrated) pFREM30 plasmid. The expected amplicon sizes are approximately 2,600 and 600 bp, respectively. The amplicons obtained were sent for sequencing (Sanger) to further confirm sequence integrity following the expected homologous recombination- mediated integration event. Sequences of used primers are listed in Table 2.

### Adhesion of *B. bifidum* PRL2010 to HT29- MTX cells

Bifidobacterial adhesion to HT29- MTX cells was assessed as previously described (Rizzo et al., 2024). Briefly, human colon carcinoma- derived mucin- secreting goblet HT29- MTX cells were cultured in Minimum Essential Medium (MEM) with high glucose (4.5 g/L). The medium was supplemented with 10% fetal bovine serum (FBS), 4 mM glutamine, 100 μg/mL streptomycin, 100 U/mL penicillin, and 10 mM HEPES. For the experiment, HT29- MTX cells were seeded on microscope cover glasses previously settled in 10-cm^2^ Petri dishes. Cells were carefully washed twice with PBS before the addition of bacterial cells. *B. bifidum* PRL2010 wild type (wt) and mutants were grown as previously described until a concentration of 1.00E + 08 cells mL^–1^ was reached. The strains were then centrifuged at 5,000 × *g* for 8 min, resuspended in PBS, and incubated with HT29- MTX cells. After 1- h incubation at 37°C, cells were washed twice with 2 mL of PBS to remove unbound bacteria. The cells were then fixed with 1 mL of methanol and incubated for 8 min at room temperature. Finally, the cells were stained with 1.5 mL of Giemsa stain solution (1:20 in PBS) (Sigma Aldrich, Milan, Italy) and incubated in the dark for 30 min at room temperature. After two washes with 2 mL of PBS, cover glasses were removed from the Petri plate, mounted on a glass slide, and examined using a Leica DM 1000 phase contrast microscope (objective: 100X/1.4 oil). The adherent bacteria in 20 randomly selected microscopic fields were counted and averaged, as previously described (Rizzo et al., 2023). The proportion of bacterial cells that remained attached to HT29- MTX cells was determined to reflect the extent of specific host–microbe interactions. The adhesion index is calculated as the average number of bacterial cells counted on 12 random spots/(100 HT29- MTX cells) (Guglielmetti et al., 2008).

### Macrophage activation by *B. bifidum* PRL2010

*B. bifidum* wild type and mutant strains were grown in MRS broth at 37°C under anaerobic conditions until they reached the exponential growth phase (0.6 < OD600 nm < 0.8). Bacterial cells were counted using a Thoma cell counting chamber (Herka), diluted to a final concentration of 2.5 × 10^5^ cells/mL, washed in PBS, and resuspended in 5 mL of antibiotic-free RPMI supplemented with 10% human AB^+^ serum (Sigma-Aldrich) and 2 mM glutamine (Gibco/Invitrogen). Human acute monocytic leukemia THP-1 cells were obtained from the Cell Bank of the Istituto Zooprofilattico Sperimentale della Lombardia ed Emilia-Romagna (Brescia, Italy). These cells are widely used *in vitro* to obtain a macrophage-like phenotype after differentiation with phorbol myristate acetate (PMA). THP-1 cells were maintained in RPMI supplemented with 10% human AB^+^ serum (Sigma-Aldrich) and 2 mM glutamine (Gibco/Invitrogen) at 5% CO_2_ and 37°C. Two days before the experiments, THP-1 monocytes were collected into 15 ml tubes and incubated for 3 h at 37°C under gentle rotation in the presence of PMA (100 nM) in complete culture medium. Thereafter, the cells were pelleted, washed three times in PBS, and seeded at a density of 3 × 10^5^ cells/cm^2^ in a 24-wells culture plate. Differentiated THP-1 cells were used for co-culture with bacteria. Thereafter, supernatants were collected after 4 h of exposure and stored at -20°C, while human/bacterial cells were preserved at −80°C until they were processed. Cells were subjected to RNA extraction as previously described (Alessandri et al., 2023; Tarracchini et al., 2023). Briefly, total RNA from human cell was extracted by adding 350 μL of RLT buffer from the RNeasy Mini Kit (Qiagen, Germany) following the manufacturer’s instructions. Subsequently, reverse transcription to cDNA was performed with the iScript Select cDNA synthesis kit (Bio-Rad Laboratories) using the following thermal cycle schedule: 5 min at 25°C, 30 min at 42°C, and 5 min at 85°C. Cytokine expression was assessed by qRT-PCR using the following primers: TNF-α_Fw (5′ ATGAGCACTGAAAGCATGATCC 3′), TNF-α_Rv (5′ GAGGGCTGATTAGAGAGAGGTC 3′. Amplifications were carried out on a CFX96 system (BioRad, CA, United States) using the PowerUp SYBR Green Master Mix (Applied Biosystem, United States) applying the following protocol: one cycle of 50 °C for 2 min, 95 °C for 2 min followed by 40 cycles of 95 °C for 15 s and 62°C. In each run, negative controls (no DNA) were included. Gene transcription was normalized relative to housekeeping genes: β-globin and Ribosomal Protein L15 (RPL15). The primers used are: β-globin_Fw-5′′ ACACAACTGTGTTCACTAGC 3′—β-globin_Rv-5′ CAACTTCATCCACGTTCACC 3′—and RPL15_Fw-5′ GCAGCCATCAGGTAAGCCAAG 3′ -RPL15_Rv-5′ AGCGGACCCTCAGAAGAAAGC 3′. The results regarding mRNA expression levels were reported as fold-of-induction in comparison with the control, i.e., THP-1 cells not exposed to bifidobacterial strains. The Human Procarta Plex Seven-plex (Thermo Fisher Scientific) was customized for the titration of TNF-α. Cytokine detection was performed following manufacturer’s recommendation and quantification was carried out with the Bioplex MAGPX^®^ (BioRad Instrument, Hercules, CA, United States).

### In vivo experimental design

For the *in vivo* trial, 5-week-old Wistar rats were used, as described by Rizzo et al. (2024). Briefly, five male and five female rats were treated with *B. bifidum* PRL2010 wild type at a dose of 10E + 09 cells/mL for a period of 2 weeks. Bacterial strains were administered using a sterile syringe in a 2% sucrose solution. This method of administration was introduced during the first week of the *in vivo* trial (referred to as the adaptation period) in order to familiarize the animals with the procedure before the actual treatment phase began. Fecal samples were collected at 7, 14, and 21 days following the initial administration, following the protocol previously described (Rizzo et al., 2024).

### Evaluation of *B. bifidum* PRL2010 TA genes transcription by qRT-PCR

To evaluate whether the exposure to the rodent gut epithelium plays a role in modulating transcription levels of TA biosynthesis-associated genes in *B. bifidum* PRL2010, RNA extraction was performed from the collected fecal samples of the rats enrolled in the *in vivo* trial and treated with *B. bifidum* PRL2010, as previously described. Starting from 0.2 g of stool sample (Milani et al., 2020), the extracted RNA was subjected to reverse transcription to obtain cDNA with the iScript Select cDNA synthesis kit following the supplier’s instruction (BioRad Laboratories). Then, the cDNA was exploited as a template to evaluate gene expression of TA-associated genes through qRT-PCR using *B. bifidum* PRL2010-specific primers (Table 3). qRT-PCR was performed using the PowerUp SYBR Green Master Mix (Applied Biosystem, United States) on a CFX96 system (BioRad, CA, United States). PCR products were detected with SYBR green fluorescent dye and amplified according to the following protocol: one cycle of 50 °C for 2 min, 95 °C for 2 min followed by 40 cycles of 95 °C for 15 s and 60°C for 1 min. In each run, negative controls (no DNA) were included. Gene transcription was normalized relative to housekeeping genes as previously described (Turroni et al., 2011).

**TABLE 3:** *B. bifidum* PRL2010-specific primers for the amplification of TA-associated genes.

Primer name	Sequence forward 5′–3′	Sequence reverse 5′–3′
BBPR_0699	GGACAAGCGCTT CGACATTC	TAGCCGACCGACT TGAAACC
BBPR_0702	ACAGTCTGTTCG CCTTGTCG	CCAAGCCCAAGC AGTTTCTG

### Ethical statement

All experimental procedures and protocols involving animals were approved by the Italian legislation on animal experimentation (D.L. 04/04/2014, n. 26, authorization n° 370/2018- PR) and conducted in accordance with the European Community Council Directives dated September 22, 2010 (2010/63/UE). The procedures were previously described in Rizzo et al., 2024 (Rizzo et al., 2024).

### Statistical analysis

Differences in bacterial adhesion to HT29 cells were evaluated by non-parametric independent-samples Kruskal–Wallis Test analysis using IBM SPSS Statistics for Windows.

## Results and discussion

### Distribution of genes predicted to be associated with teichoic acid biosynthesis in the genus *Bifidobacterium*

Although various studies have demonstrated that TAs produced by Gram-positive bacteria are directly involved in the microbe-host dialogue, the distribution of genes involved in the TA biosynthesis machinery in bifidobacteria has not yet been investigated in great detail (Castro-Bravo et al., 2018; Hidalgo-Cantabrana et al., 2014b; Milani et al., 2017c; Pyclik et al., 2020; van Dalen et al., 2020).

Bifidobacterial genome screening for genes predicted to be involved in TA biosynthesis revealed the presence of a single gene cluster as well as conserved genes scattered throughout bifidobacterial genomes, suggesting that only a few genetic sequences involved in TA production are widespread among *Bifidobacterium* species (Figure 1 and Supplementary Table S2). In detail, only two conserved TA biosynthesis-associated genes were identified in nearly all analyzed genomes, represented by *tag*O and a gene predicted to encode a lipoteichoic acid (LTA) synthase. Specifically, *tag*O had previously been characterized in *Bacillus subtilis* to encode an enzyme responsible for catalyzing the first step in TA biosynthesis, an UDP-N-acetylglucosamine:undecaprenyl-P N-acetylglucosaminyl 1-P transferase (Soldo et al., 2002). However, identification of these genes does not point to the existence of a suitable genomic repertoire able to synthesize TA in bifidobacteria.

In contrast, genes of the *tag* cluster were observed among strains belonging to the *Bifidobacterium amazonense, B. bifidum, Bifidobacterium catulorum, Bifidobacterium leontopitheci, Bifidobacterium pluvialisilvae*, *Bifidobacterium primatium*, and *Bifidobacterium pullorum* species (Figure 1). The *tag* cluster was mainly identified by the presence of two *tag*B genes predicted to encode the teichoic acid biosynthesis protein B, also known as CDP-glycerol glycerophosphotransferase, which is responsible for the polymerization of the main chain of TA in *Bacillus subtilis* (Bhavsar et al., 2005). Furthermore, the *tag* cluster was also characterized by the presence of two genes predicted to encode for an ABC transporter system named *tag*G and *tag*H, also well characterized in *Bacillus subtilis* (Lazarevic and Karamata, 1995), responsible for membrane translocation of TA precursors. Finally, based on the different bifidobacterial species, three to five genes were also located in between the four genes above reported. Among them, we found the conserved *isp*D gene, which encodes a 2-C-methyl-D-erythritol 4-phosphate cytidylyltransferase. This enzyme is part of the methylerythritol phosphate (MEP) pathway and contributes to the synthesis of a lipid carrier essential for the translocation of cell wall precursors, including teichoic acids, across the bacterial membrane (Burd et al., 2013; Mendillo et al., 2012). While *ispD* is not directly involved in TA polymerization, its metabolic role is likely to support TA biosynthesis by supplying key intermediates required for precursor transport. Interestingly, among the bifidobacterial species that possess the *tag* cluster, only *B. bifidum* is of human origin, representing a key microbial species of the infant gut microbiota (Milani et al., 2017b; Figure 1 and Supplementary Table S2). These *in silico* findings suggest that, among the different bifidobacterial species inhabiting the human gut, the *tag* cluster of *B. bifidum* may provide to this species with putative advantages in colonizing and persisting in the human intestinal ecological niche. Thus, this observation guided us in implementing multiple *in vitro* experiments to evaluate whether the disruption of some of the genes putatively involved in TA production affects the ability of *B. bifidum* to adhere to the intestinal epithelium.

### *In vitro* impact of bifidobacteria-host interaction on teichoic acid production

**FIGURE 2:**
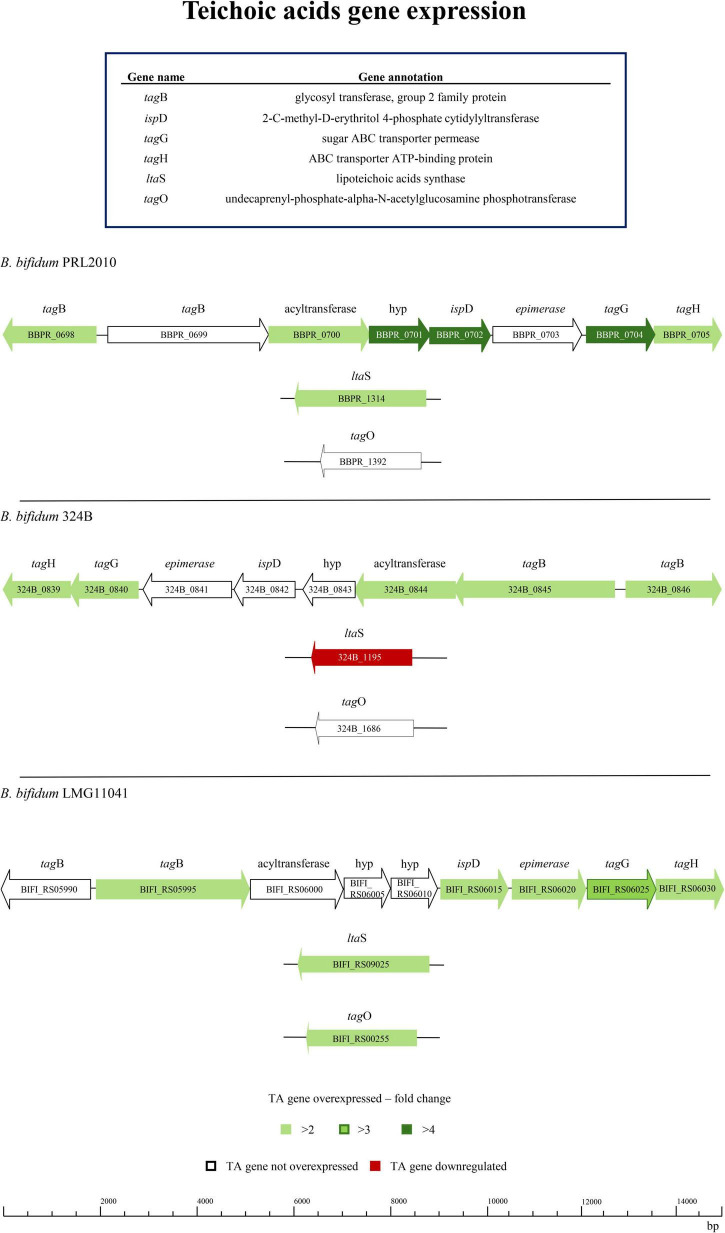
Transcriptomics analysis of three *B. bifidum* strains exposed to human cell monolayers. The genetic maps depict the distribution and the up- or down-regulation of TA-associated genes of *B. bifidum* strains after having been in contact for 4 h with a Caco-2/HT29-MTX human cell monolayer when compared to the control.

Bifidobacterial ability to persist in the intestinal ecological niche is influenced by the production of different cell wall structures (Castro-Bravo et al., 2018; LeBlanc et al., 2013; Ligthart et al., 2020; Milani et al., 2017c; Robinson et al., 2010; van Dalen et al., 2020). Therefore, it is possible that a different genetic repertoire for TA production affects bifidobacterial colonization and persistence of the infant intestine. In this context, *in silico* analyses showed a restricted distribution of genes associated with the *tag* cluster for TA biosynthesis among bifidobacterial species. Since the only bifidobacterial taxon of human origin harboring the identified *tag* cluster is *B. bifidum*, evidence of events of exogenous DNA acquisition were sought. The surrounding area of the *tag* cluster of the *B. bifidum* strains analyzed does not show the presence of transposases, which suggests that the *tag* cluster of *B. bifidum* had not been acquired by recent horizontal gene transfer. These findings seem to underline the specificity of TA-encoding genes in *B. bifidum* (Peters et al., 2014). To assess whether differences in TA gene transcription occur when different strains belonging to the same species are exposed to the human host, three *B. bifidum* strains were seeded on Caco-2/HT29-MTX cell monolayer for 4h, as previously described (Alessandri et al., 2023; Fontana et al., 2022; Longhi et al., 2024; Tarracchini et al., 2023). Specifically, three strains predicted to encode the same genetic repertoire for TA biosynthesis, were selected for *in vitro* transcriptome analysis (Table 1 and Figure 2). Changes in gene transcription between each bifidobacterial strain, placed in contact with human cells, and its corresponding counterpart not exposed to the human cell monolayer, i.e., bacterial control cells, were assessed by RNAseq. Subsequently, only genes showing a fold-change of ≥ 2 in combination with a *p*-value of ≤ 0.05, calculated through correction for multiple comparisons using the False Discovery Rate (FDR) procedure, were considered as significantly differentially expressed between the two conditions. Interestingly, in depth scrutiny of RNAseq data highlighted clear intra-species differences in transcription of genes putatively involved in TA production when bifidobacterial strains were exposed to human cell line monolayers (Figure 2 and Supplementary Table S3). Specifically, *B. bifidum* strain PRL2010 highly responded to contact with human epithelial cells by modulating transcription of their TA-associated genes. However, while PRL2010 showed increased transcription of the highest number of predicted TA genes (7 out of 10), with the remaining genes maintaining the same transcription level with respect to the control (Figure 2), the other *B. bifidum* assessed strains, i.e., 324B and LMG11041, showed significantly increased transcription of a smaller number of genes (five or seven out of 10 or 11 genes, respectively) compared to PRL2010 when exposed to human cell lines. Additionally, for *B. bifidum* strain 324B transcription of just a single gene associated with TA production was shown to be down-regulated (Figure 2), thus suggesting that differential transcriptional regulation of these genes occurs among *B. bifidum* strains. Overall, these results highlight that intra-species differences can be observed in the transcriptional profiles of predicted TA biosynthesis genes among strains of *B. bifidum*, suggesting that different strains employ distinct host-interaction strategies to ensure epithelium adhesion, gut colonization and microbe-host communication.

### Teichoic acids and adhesion of bifidobacteria to HT29-MTX cells monolayer

One of the functions of TAs described in scientific literature is that they promote adhesion to eukaryotic cells (Keinhörster et al., 2019). Therefore, to assess whether TA production impacts on adhesion abilities of *B. bifidum* PRL2010, which represents the prototype of the *B. bifidum* species (Fontana et al., 2022), to human mucus- secreting HT29- MTX cells, three different mutants in predicted TA-associated genes were employed. The selected genes, corresponding to locus tags BBPR_0699, BBPR_0702, and BBPR_1314, were selected based on their putative involvement in TA biosynthesis. Specifically, BBPR_0699 and BBPR_0702 are located within a previously described species-specific gene cluster conserved among *B. bifidum* strains and potentially implicated in TA production (Colagiorgi et al., 2015). BBPR_0699 was selected because of it is predicted as a homolog of *tagB*, which is a gene encoding CDP-glycerol glycerophosphotransferase involved in TA polymerization in *Bacillus subtilis*. BBPR_0702 was included because, unlike other genes in the same cluster, it does not encode a transporter or permease, indicating a potentially distinct biosynthetic role. In addition, BBPR_1314 was chosen based on its annotation as the *B. bifidum* homolog of *ltaS* from *Staphylococcus aureus*, which encodes a lipoteichoic acid synthase (Colagiorgi et al., 2015).

**FIGURE 3:**
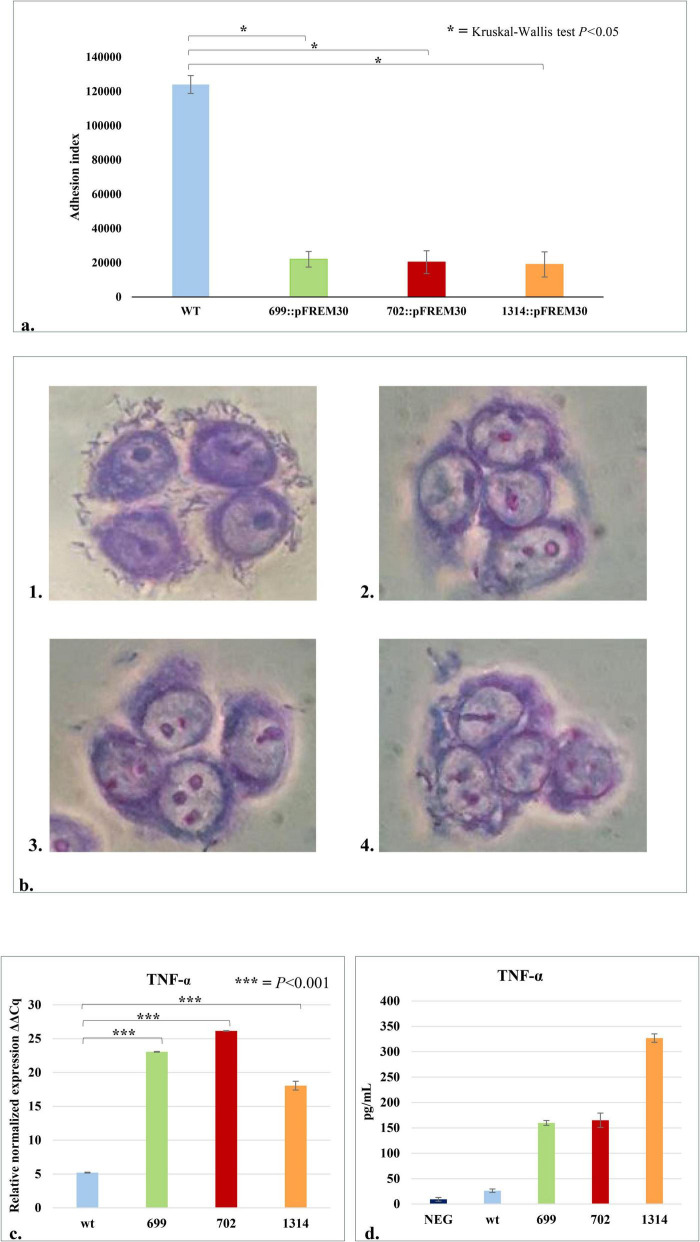
Interaction of *B. bifidum* PRL2010 cells with human host cells. The wt and the three mutant strains of *B. bifidum* PRL2010 were placed in contact with HT29-MTX cell monolayers to test their adhesion abilities and with macrophage-like cells, i.e., THP-1, to detect differences in the production of TNF-α by human cells. **(a)** The adhesion performances of *B. bifidum* PRL2010 wt and mutants with a disruption in one of the TA-associated genes, i.e., *B. bifidum* 699:pFREM30, 702:pFREM30, 1314:pFREM30, to HT29-MTX cell monolayers, expressed as the adhesion index. The vertical bars indicate standard deviations (*n* = 12), and the asterisks indicate statistical differences among adhesion indexes of the mutants and wt based on Kruskal-Wallis Test *p* < 0.05. **(b)** Light microscopy images of Giemsa-stained HT29-MTX cells incubated with *B. bifidum* PRL2010. The bifidobacterial strains shown in each image are: (1) *B. bifidum* wt, (2) *B. bifidum* 699:pFREM30, (3) *B. bifidum* 702:pFREM30 and (4) *B. bifidum* 1314:pFREM30. **(c)** The transcriptome levels of TNF-α produced by the host cells when exposed to the wt and the three mutant strains. The y-axis represents the normalized expression level (ΔCt) according to CFX96 Bio-Rad software relative to the control (THP-1 cells without any contact with bacterial cells). The vertical bars indicate standard deviations (*n* = 3) and the asterisks indicate statistical differences among TNF-α produced by immune cells placed in contact with the mutants and wt (Bonferroni *Post Hoc p* < 0.001). **(d)** Reports the amount of TNF-α, expressed in pg/ml of culture supernatant, produced by THP-1 cells after or without the contact with bacteria (negative). The mean results of two independent assays are shown. The asterisks indicate the statistical significance level of the results: *p-value < 0.05, ***p-value < 0.001.

The *B. bifidum* PRL2010 insertion mutants for genes BBPR_0699, BBPR_0702 -belonging to the *tag* cluster —and BBPR_1314 encoding a CDP-glycerol glycerophosphotransferase family protein, a 2-C-methyl-D-erythritol 4-phosphate cytidylyltransferase and a LTA synthase protein, show a significant reduction in the adhesion index (average number of bacterial cells/100*HT29- MTX cells) to HT29- MTX cells (Figure 3a). Indeed, the adhesion index values of the three mutants, *B. bifidum* 699:pFREM30 (adhesion index 22,000 ± 4,554), *B. bifidum* 702:pFREM30 (adhesion index 20,333 ± 6,655) and *B. bifidum* 1314:pFREM30 (adhesion index 19,000 ± 7,333) were significantly (Kruskal-Wallis Test *P* < 0.05) lower than that obtained for *B. bifidum* PRL2010 wt (adhesion index 124,000 ± 5,221) (Figures 3a,b). These results suggest that the inactivation of predicted TA-associated genes causes a decrease in the adhesion index of bifidobacterial cells to human intestinal cells, strengthening the hypothesis of a direct involvement of these genes in the production of cell wall structures such as TAs, which may be involved in the colonization and engraftment of bifidobacterial cells in the human gut.

### Immunomodulatory activities exerted by TA of *B. bifidum* PRL2010 cells

The importance of various structures produced by bifidobacterial cells in driving an interaction with the human immune system has been reported previously (Arrieta et al., 2018; Henrick et al., 2021; Hidalgo-Cantabrana et al., 2014a; Kumar et al., 2016; Lee et al., 2021; Longhi et al., 2020; Rhoads et al., 2018). In the current study, an experiment with human acute monocytic leukemia THP-1 cells, differentiated to exhibit a macrophage-like phenotype, and PRL2010 was performed to evaluate whether TA encoded by *B. bifidum* PRL2010 influences the host innate immune response. In detail, *B. bifidum* PRL2010 wt and the three predicted TA mutants were placed in contact with human cells in order to investigate the secretion by the immune cells of a pro-inflammatory cytokine, i.e., TNF-α. Interestingly, the expression levels, determined by qRT-PCR, of TNF-α considered for this experiment were statistically higher in the THP-1 cells in contact with the mutant strains compared to the ones in contact with the wild type strain (Bonferroni *Post Hoc p* < 0.001) (Figure 3c). These data are further confirmed by the quantification of the cytokine through ELISA assay, which highlights differences in TNF-α production between human cells in contact with PRL2010 wt and mutants (Figure 3d). Notably, even though the number of the samples does not allow to perform a robust statistical test, the observational results highlight that the production of TNF-α is higher in human cells interacting with the three mutants compared to the contact with PRL2010 wt, pointing out the difference between strains whose TA production is different. The study of the immune response was focused on the production of TNF-α, which is among the first cytokines produced after contact with the antigen that can lead to a severe systemic inflammation if not under control of the immune mechanisms of the host (Fink et al., 2007). These results clearly indicate that the predicted PRL2010 TA-biosynthesis genes are implicated in regulating the enhanced production of TNF-α by the host, as previously reported for TA of other bacteria (Kaji et al., 2010; Matsuguchi et al., 2003; Volz et al., 2018). This immunomodulatory effect may be mediated by the recognition of TA as microbe-associated molecular patterns (MAMPs) by pattern recognition receptors (PRRs), particularly Toll-like receptor 2 (TLR2), leading to the activation of signaling pathways such as NF-κB and MAPK (Kawai and Akira, 2010; Shoop et al., 2001). Additionally, the specific structure and chemical modifications of TA, such as glycosylation or D-alanylation, may influence their immunogenic potential and interaction with host immune receptors (Xu et al., 2005). Furthermore, as TA molecules are also involved in bacterial adhesion to epithelial cells, their presence or absence may modulate localized immune responses at mucosal surfaces (Houni et al., 2008). It is also plausible that TAs contribute to shaping immune tolerance by affecting the activation or maturation state of antigen-presenting cells, including dendritic cells, particularly in early-life contexts where bifidobacteria such as *B. bifidum* are prevalent (Hooper et al., 2012). These preliminary data will be crucial for future more in-depth studies investigating the host’s immune response and its regulation by TA of bifidobacteria.

### Assessment of the predicted TA-encoding gene expression levels in *B. bifidum* PRL2010 following oral administration to murine models

**FIGURE 4:**
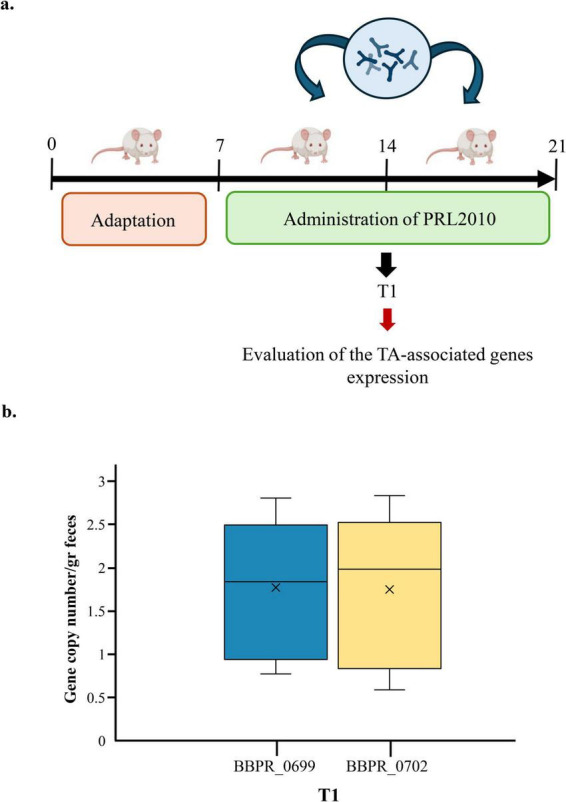
Evaluation of the expression of TA-associated genes of *B. bifidum* PRL2010 cells under *in vivo* conditions. **(a)** Represents the timeline of the experimental procedure in the murine model. **(b)** The mRNA levels of two different TA-associated genes, i.e., BBPR_0699 and BBPR_0702, in the fecal samples of rats at T1. The *y*-axis represents the gene copy numbers of *B. bifidum* PRL2010 per gram of feaces, while the *x*-axis represents the time point considered for the evaluation of genes expression.

To further corroborate our *in vitro* findings, we evaluated the potential ability of PRL2010 to overexpress genes presumed to be involved in TA production following its gut colonization. In the framework of a previous *in vivo* study, aimed at assessing the impact of *B. bifidum* PRL2010 in a rodent model, we specifically examined whether the expression of TA-associated genes was modified upon gut colonization (Rizzo et al., 2024; Figure 4a). In this study, wild type PRL2010 was administered daily at a dose of approximately 10E + 09 cells/ml, and fecal samples were collected freshly from each animal at three different time points, i.e., 7 days (T1), 14 days (T2), and 21 days (T3) after the first administration (Figure 4a). The expression levels of two key TA-encoding genes, BBPR_0699 and BBPR_0702, were assessed by qRT-PCR at T1 since at this time point the level of PRL2010 colonization was previously determined to be the highest among the three time points (Rizzo et al., 2024). These genes were selected because they belong to a conserved gene cluster across *B. bifidum* strains predicted to be involved in teichoic acid biosynthesis and were not annotated as transporters or permeases. As shown in Figure 4b, the results showed a remarkable up-regulation of these TA-associated genes in the fecal samples at T1, highlighting a clear induction of gene expression shortly after administration. To further support this finding, a global genome transcription profile of PRL2010 was previously performed both in an *in vitro* human gut model and on fecal samples from BALB/c mice following supplementation, revealing the expression of BBPR_0699 gene under both conditions (Turroni et al., 2013). Specifically, this key gene was up-regulated in the murine cecum as well as after incubation with human intestinal HT29 cells, suggesting potential interactions with its natural ecological niche (Turroni et al., 2013). Overall, these data emphasize that contact with the intestinal environment, including murine epithelium, is the factor triggering the production of TA by PRL2010, thus corroborating our *in vitro* data showing the pivotal importance of these cell wall structures in the interaction with the host.

## Conclusion

Since bifidobacteria are common inhabitants of the human gut and are generally considered to confer host health benefits, various studies have in recent decades investigated the molecular background responsible for the interaction of these bacteria with the human host (Milani et al., 2017c; Reid, 2023; Salazar et al., 2016). Nonetheless, although TAs are among the various cell wall structures identified as putative key factors for host-bifidobacteria interaction (Turroni et al., 2021), little is known about their genetic diversity within the *Bifidobacterium* genus and about regulation of TA production. Our study reveals a limited and variable genetic distribution of predicted TA-associated genes throughout the examined bifidobacterial genomes, suggesting functional diversity among species in their ability to produce these key molecules. However, we identified a specific gene cluster, the *tag* cluster, present exclusively in certain species including *B. bifidum*, which may represent a distinctive factor in host interaction. Such *in silico* data will be further corroborated by follow up studies aimed at the biochemically characterization of the presumed TA molecules both in the wild type and in the mutant strains, as previously reported for other microorganisms (Fiedler, 1988; Lu et al., 2009; Morath et al., 2001). Furthermore, transcriptomic analyses demonstrated that contact with human epithelial cells induces differential expression of TA genes, supporting the hypothesis that these compounds respond to environmental and host-derived stimuli within the intestinal niche. Notably, *in vitro* experiments with PRL2010 mutants confirmed the functional role of TAs in the adhesion to intestinal cells and modulation of immune responses, highlighting a potential mechanism through which bifidobacteria promote persistence and beneficial effects in the human gut. Such results have been found both in other purported health-promoting microorganisms as well as pathogenic bacteria, corroborating the presumed involvement of bifidobacterial TAs in adhesion to human epithelial cells (Gusils et al., 2002; Lehmann et al., 2024). The observation of increased TA gene expression in a murine model strengthens the notion of host-dependent regulation of TA production and paves the way for future studies to elucidate the underlying regulatory mechanisms. Overall, these findings emphasize the importance of TAs as key mediators in host–microbiota communication, with promising clinical and biotechnological implications for modulating intestinal and immune health. However, it is important to note that teichoic acids are not the only bacterial surface structures involved in host interactions; other components such as pili and exopolysaccharides also play crucial roles in microbe-host communication. While this study focused specifically on teichoic acids, we acknowledge the complexity of these interactions and will plan further research to explore the contribution of other surface molecules. Clinical validation will be crucial to translate these insights into effective therapeutic applications.

## Data Availability

The original contributions presented in the study are included in the article/Supplementary material, further inquiries can be directed to the corresponding author.

## Footnotes

1
https://broadinstitute.github.io/picard/
